# Risk Factor Analysis for Long-Term Graft Survival Following Pediatric Kidney Transplantation: The Importance of Pretransplantation Time on Dialysis and Donor/Recipient Age Difference

**DOI:** 10.3390/jcm12227014

**Published:** 2023-11-09

**Authors:** Marios Marcou, Matthias Galiano, Anja Tzschoppe, Katja Sauerstein, Sven Wach, Helge Taubert, Bernd Wullich, Karin Hirsch-Koch, Hendrik Apel

**Affiliations:** 1 Clinic of Urology and Pediatric Urology, University Hospital Erlangen, Friedrich-Alexander-Universität Erlangen-Nürnberg, 91054 Erlangen, Germany; sven.wach@uk-erlangen.de (S.W.); helge.taubert@uk-erlangen.de (H.T.); bernd.wullich@uk-erlangen.de (B.W.); karin.hirsch@uk-erlangen.de (K.H.-K.); hendrik.apel@uk-erlangen.de (H.A.); 2 Clinic of Pediatrics and Adolescent Medicine, University Hospital Erlangen, Friedrich-Alexander-Universität Erlangen-Nürnberg, 91054 Erlangen, Germany; matthias.galiano@uk-erlangen.de (M.G.); anja.tzschoppe@uk-erlangen.de (A.T.); katja.sauerstein@uk-erlangen.de (K.S.); 3 Transplant Centre Erlangen-Nürnberg, University Hospital Erlangen, Friedrich-Alexander-Universität Erlangen-Nürnberg, 91054 Erlangen, Germany

**Keywords:** kidney transplantation, children, graft survival, age difference, renal dialysis

## Abstract

Recognizing risk factors that may negatively affect long-term graft survival following pediatric kidney transplantation is a key element in the decision-making process during organ allocation. We retrospectively reassessed all cases of pediatric kidney transplantation performed in our center in the last 20 years with the aim of determining baseline characteristics that could be identified as prognostic risk factors for long-term graft survival. Between 2001 and 2020, a total of 91 kidney transplantations in children under the age of 18 years were undertaken in our center. Early graft failure was observed in six of the 91 patients (7%). The median follow-up of the remaining 85 children was 100 months, and the overall kidney graft survival rates at 5, 10, 15 and 20 years were 85.2%, 71.4%, 46.0% and 30.6%, respectively. Small children with a body surface area of <1 m^2^ were significantly associated with better long-term graft survival outcomes, while adolescents aged more than twelve years showed poorer graft survival rates than younger children. Body surface area of the recipient of ≥1 m^2^, pretransplantation duration of the recipient on dialysis ≥18 months, hemodialysis prior to transplantation and donor/recipient age difference of ≥25 years were significantly associated with poorer long-term graft survival.

## 1. Introduction

End-stage renal disease (ESRD), the irreversible damage of the kidneys leading to the necessity of renal replacement therapy, is a major public health issue, and the estimated incidence of ESRD in children across the world was nine per million of the age-related population (4–18 years) in 2008 [1], while according to data from the United States Renal Data System (USRDS), 12.9 per million population of children under the age of 18 were diagnosed with ESRD in the United States in 2017 [2]. The most prevalent primary causes of ESRD in children are congenital anomalies of the kidneys and urinary tract (CAKUT), nephrotic syndrome, glomerulonephritis and hereditary nephropathies [1,3]. Congenital disorders are responsible for about two-thirds of all cases of ESRD in developed countries, while acquired causes predominate in developing countries [1]. ESRD during childhood may lead to developmental delay, growth retardation and bone loss and is associated with long durations of hospitalization of the affected children [4,5]. Furthermore, the need for dialysis can have a significant psychological impact on children as well as their caregivers [6]. Of even greater importance is the high rate of mortality of children under sustained dialysis, i.e., a 10.5% mortality in a 5-year interval [7].

The best and most effective renal replacement therapy for children with ESRD is kidney transplantation [8,9]. Pediatric kidney transplantation reflects only a small percentage of overall kidney transplantations, and the number of pediatric kidney transplantations performed in each center every year, compared to adult kidney transplantations, are limited. From an overall of about 40,000 kidney transplantations performed in Europe and in the United States each year, only around 1300 kidney transplantations are performed in children [10]. A series of factors, such as age and comorbidities of both the donor and recipient, sex mismatch between the donor and the recipient, body surface area (BSA) of the recipient, pretransplantation time on dialysis, modality of dialysis prior to transplantation, immunological profile, human leukocyte antigen (HLA)-matching between the donor and the recipient, and duration of cold ischemia of the graft, have been suggested to affect long-term graft survival following pediatric kidney transplantation [11,12,13,14,15]. In this study, we retrospectively reassessed all cases of pediatric kidney transplantation in our center with the aim of determining characteristics that could be identified as prognostic risk factors for long-term graft survival.

## 2. Materials and Methods

A retrospective review of all cases of kidney transplantation in recipients aged less than 18 years performed in our center between 2001 and 2020 was conducted. Following the identification of cases using our hospital database, a thorough review of the electronic medical files of the patients was undertaken, and the following baseline characteristics were documented: source of the donor organ (living or deceased), age and sex of the recipients, age and sex of the donor, weight and height of the recipients at transplantation, number of transplantations (primary or retransplantation), pretransplantation duration of dialysis (not including previous time on dialysis in cases of retransplantation), modality of dialysis performed prior to transplantation (peritoneal dialysis or hemodialysis), number of HLA mismatches and duration of cold ischemia of the graft. The primary outcome of this study was graft survival, determined as the need to restart dialysis, transplantectomy or retransplantation. Baseline characteristics were compared by chi-squared analysis, and graft survival estimates were analyzed using the Kaplan–Meier method. Continuous covariates were stratified for further analyses. The basis for defining the cutoff values was the population median. Nevertheless, if the distribution of continuous variables gave a clear indication of a biphasic distribution of data values, the cutoff value was modified based on the kernel density distribution in order to reflect the real-life distribution of data values. To determine the independent predictive variable for graft loss, significant factors in the univariate analysis were fitted into a multivariate Cox regression analysis. Statistical analysis was performed using the R statistical computing program (V4.2.1; R Foundation for Statistical Computing, Vienna, Austria) with statistical significance set at *p* < 0.05 [16].

## 3. Results

Between 2001 and 2020, a total of 91 kidney transplantations in children under the age of 18 years were undertaken in our center. Eighty-five of them (93%) originated from a deceased donor (DD), and only six (7%) originated from a living donor (LD). The cause of death of the DD was always brain death, and no DD kidneys following circulatory death (nonheart-beating donors) were accepted. There were thirteen (14%) cases of retransplantation as a consequence of graft failure following transplantation at a younger age.

Following transplantation, early graft failure was observed in 6 of the 91 cases (7%). There were two cases of acute graft rejection, two cases of graft thrombosis, one case of sepsis with formation of renal/perirenal abscess, and one case with no graft function following transplantation. Four of the six abovementioned cases were treated with immediate transplantectomy in the days following transplantation. One patient died as a result of a hyperacute rejection episode before removal of the graft. In the case where no graft function was observed, transplantectomy was performed one year later. All cases of early graft failure were transplantations from DD, and only one was a retransplantation.

Long-term graft survival was assessed in the remaining 85 children. The median follow-up was 100 months (IQR 53–137 months). During follow-up, loss of function was reported in a total of 30 (35.3%) kidney grafts (Table 1), and the overall kidney graft survival rates at five, ten, fifteen and twenty years were 85.2%, 71.4%, 46.0% and 30.6%, respectively. It is however important to mention that of the 27 children that were transplanted more than fifteen years ago (prior to 2008), loss of function was reported in 13 of the children, 8 children were lost to follow-up and only 6 of the children were followed up for a total of more than fifteen years. Of the eight children who were transplanted more than twenty years ago (prior to 2003), loss of function was reported in four of the children, two of the children were lost to follow-up and only two of the children were followed-up for a total of more than twenty years. No death of a recipient with a functioning graft was documented during follow-up.

**Figure 1:**
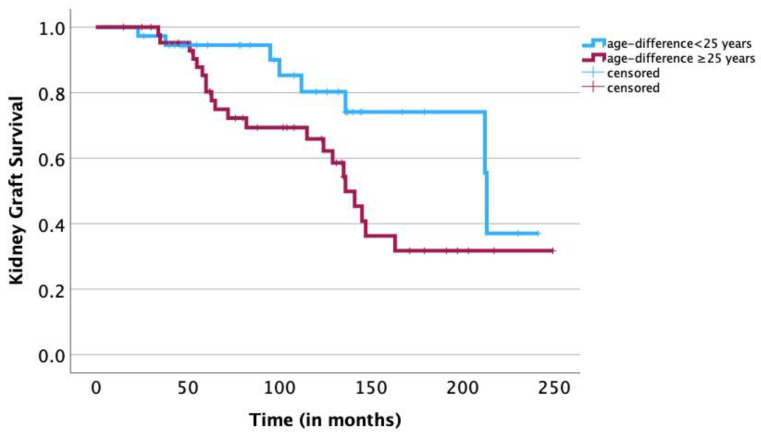
Kaplan–Meier survival analysis: association of age difference between donor and recipient with long-term survival of kidney graft. A univariate log-rank analysis shows a significant association between a large age difference between donor and recipient (≥25 years) and a shorter survival time of the kidney graft compared to patients with a smaller age difference <25 years (*p* = 0.04). Graft loss of pediatric recipients with a donor/recipient age difference of ≥25 years appeared after a median duration of approximately six years (IQR 57–136 months), and long-term graft survival of these patients at five, ten, fifteen and twenty years was 85.3%, 65.9%, 31.7% and 31.7%, respectively, compared to 95.5%, 80.3%, 74.1% and 37.0% of recipients with a donor/recipient age difference of <25 years.

**Figure 2:**
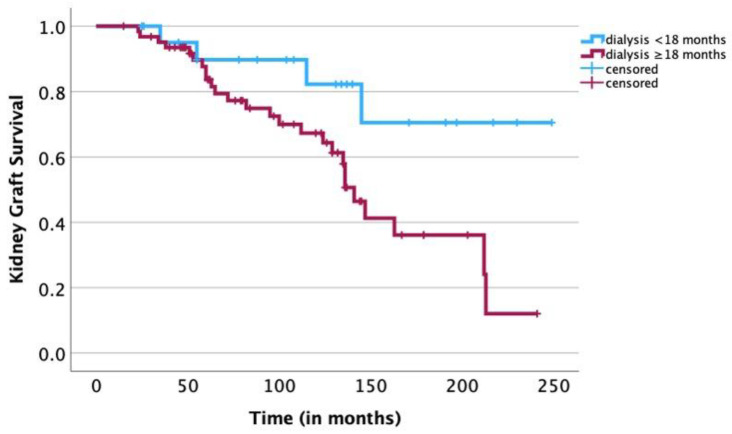
Kaplan–Meier analysis: association of duration of pretransplantation time of dialysis with long-term survival of kidney graft. A univariate log-rank analysis showed a significant association between a longer pretransplantation duration of dialysis (≥18 months) and a shorter survival time of the kidney graft compared to patients with a shorter pretransplantation duration of dialysis (<18 months; *p* = 0.02).

**Figure 3:**
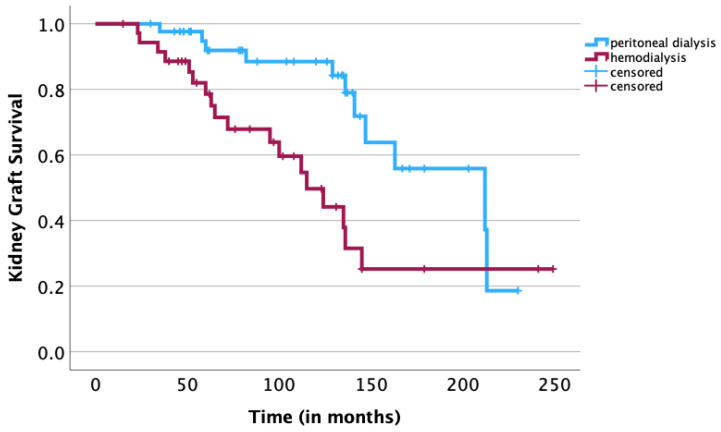
Kaplan–Meier analysis: association of the modality of dialysis undergone by the recipients preceding the kidney transplantation with long-term survival of kidney graft. A univariate log-rank analysis showed a significant association between hemodialysis and a poorer kidney graft survival rate (*p* < 0.01).

**Figure 4:**
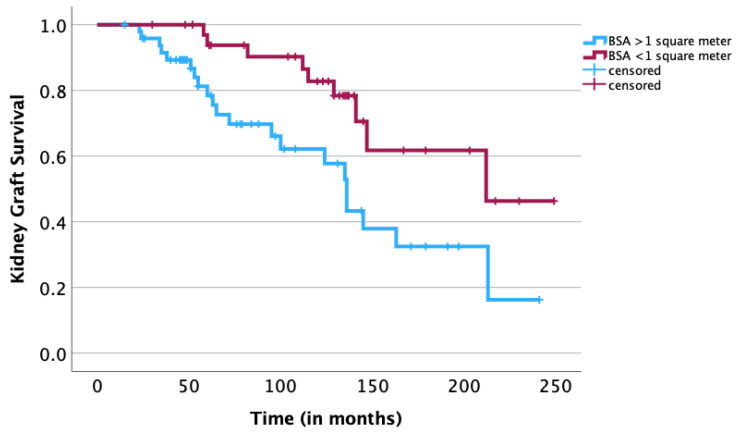
Kaplan–Meier analysis: association of body surface area (BSA) with long-term survival of kidney graft. A univariate log-rank analysis showed a significant association between a smaller BSA (<1 m^2^) and a longer survival time of the kidney graft (*p* < 0.01).

A univariate survival analysis revealed a negative statistical significance of the following risk factors: age of the recipient ≥ 12 years (*p* = 0.01), age of the donor ≥ 35 years (*p* = 0.03), donor/recipient age difference of ≥25 years (*p* = 0.04) (Figure 1), a pretransplant duration of dialysis of ≥18 months (*p* = 0.02) (Figure 2), hemodialysis prior to transplantation (*p* < 0.01) (Figure 3) and the BSA of the recipient of ≥1 m^2^ (*p* < 0.01) (Figure 4). The sex of the donor, sex mismatch between donor and recipient, the number of donor/recipient HLA mismatches and the duration of cold ischemia of the graft were not shown to statistically influence long-term graft survival in our study (Table 1). A multivariate Cox regression analysis adjusted for statistically significant factors in the univariate analysis, namely donor/recipient age difference, BSA of the recipient, modality of dialysis and pretransplantation duration on dialysis confirmed the statistical significance of a donor/recipient age difference of ≥25 years (HR 3.039; *p* = 0.014) and a pretransplantation duration of dialysis of ≥18 months (HR 3.869; *p* = 0.033) (Table 2). Replacement of the donor/recipient age difference ≥ 25 years with age of the recipient ≥ 12 years and age of the donor ≥ 35 years showed a statistical significance of the age of the donor ≥ 35 years (HR 2.929; *p* = 0.016) and again confirmed the statistical significance of a pretransplantation duration of dialysis of ≥18 months (HR 3.986; *p* = 0.032). Because of the small size of our study, fitting all nine parameters (sex of the recipient, age of the recipient, sex of the donor, age of the donor, BSA of the recipient, pretransplantation duration of dialysis, modality of dialysis performed prior to transplantation, number of HLA mismatches and duration of cold ischemia of the graft) in a multivariate Cox regression model resulted in none of the abovementioned parameters showing statistical significance.

## 4. Discussion

Kidney transplantation is unequivocally the treatment of choice for ESRD, and epidemiological studies have demonstrated that children who undergo kidney transplantation live on average 30 years longer than those who remain on dialysis [17]. In addition to advantages that are universal to all renal transplant recipients regardless of age, pediatric kidney transplantation offers unique advantages to affected children. Apart from a significant increase in overall survival compared to children remaining on dialysis, pediatric kidney transplantation is followed by enhanced linear growth, better psychomotor development and social adjustment of the children, as well as a higher quality of life of the affected children and their families [6,18].

Our study has demonstrated that transplantation of small children is associated with a longer allograft survival time. Lower weight and height and consequently a lower BSA of the child recipient are significantly associated with longer graft survival (in our study, only significant in the univariate analysis) (Figure 4); these correlations have also been reported in a series of previous studies [12,13]. It is suspected that due to the usually large renal allograft mass acquired relative to the recipients’ small body size, small recipients have substantial renal transplant reserves, which could explain the significantly longer graft survival time [19]. This has also been demonstrated in adult kidney transplantation, with smaller recipients having better long-term survival outcomes when receiving grafts from taller donors [20]. Once again, teenagers, namely, children between the ages of twelve and 18 years old, were demonstrated in our study to be significantly associated with poorer graft survival time (in our study, only significant in the univariate analysis). Adolescents have been repeatedly demonstrated to have a worse long-term graft survival time among all pediatric age groups [11,12], and it is believed that post-transplantation nonadherence of teenagers to medication may play a major role in poorer outcomes [21,22].

Furthermore, our study confirmed that donor age and donor/recipient age difference play a significant role in long-term graft survival. Specifically, we showed that a donor age ≥ 35 years and a donor/recipient age difference of ≥25 years (multivariate analysis) were significantly associated with worse long-term kidney graft survival. Accordingly, advanced donor age and high differences in age between donor and recipient have long been associated with poorer outcomes following kidney transplantation [23,24,25,26]. In a 2009 study of 7,291 pediatric kidney transplantations, Dale-Shall et al., observed that kidney grafts from living donors older than 55 years exhibited significantly poorer long-term allograft survival [27]. In a 2018 study of 1,134 kidney transplantations in recipients less than 20 years of age, Trnka et al., showed an approximately 10% increase in graft loss for every ten-year increase in donor/recipient age difference [28]. In a 2020 study of 136,321 adult kidney transplantations, Lepeytre et al. reported significantly better graft survival rates from donors aged less than 40 years [13]. Other studies have demonstrated that due to progressing glomerular sclerosis, both the glomerular filtration rate and renal plasma flow decline with age, beginning at approximately age thirty [29]. Older donor kidney grafts therefore contain less nephron mass than younger grafts, and following kidney transplantation, hyperfiltration to facilitate the increased physiological and metabolic demands of the younger recipient becomes necessary. Hyperfiltration will lead to progressing kidney damage and ultimately accelerate graft failure [30]. On the other hand, in a study of 4,686 pediatric kidney transplantations, Chesnaye et al., found no effect of donor and recipient age combinations on the five-year graft-failure risk [31]. We assume that the five-year follow-up of the study was simply too short to reflect the true impact of donor/recipient age difference, which is first made evident on long-term follow-up. In our study, after a median follow-up of 100 months, graft loss of pediatric recipients with a donor/recipient age difference of ≥25 years appeared after a median duration of approximately six years (Figure 1).

Of the 91 pediatric kidney transplantations that were performed in our center between 2001 and 2020, the number of HLA mismatches exceeded four mismatches in only four cases, with one being a transplantation from a living donor. Similarly, the cold graft ischemia time exceeded 24 h in only three cases. This may explain why the number of HLA mismatches or the duration of cold ischemia time of the kidney graft were not shown to influence long-term graft survival in our study. Despite several studies reporting both factors to be important in influencing long-term graft survival [32,33,34], the importance of HLA matching has been postulated in some studies to be less important in the current era of more powerful immunosuppression [11,35]. However, although variations in cold ischemia time in a timeframe of less than 24 h also did not significantly influence long-term outcomes in our study, a prolonged cold-ischemia time is undoubtedly a negative predictive factor and should be avoided [36,37,38]. Furthermore, although some studies have demonstrated a significant association between donor and recipient sex on kidney allograft survival in pediatric transplant recipients, showing that male-donor/male-recipient transplantations are associated with the highest survival rates [39], our study did not reveal any significant association between sex of the donor or donor/recipient sex mismatch to kidney graft survival and further larger studies are required to clarify the role of donor-sex in pediatric kidney transplantation.

Concerning pretransplantation duration on dialysis, our study confirms that time on dialysis impacts long-term graft survival. We showed that a duration of ≥18 months on dialysis was significantly associated with poorer long-term graft survival (multivariate analysis) (Figure 2). In a study of 7527 pediatric kidney recipients, Amaral et al. also demonstrated that when compared with children undergoing preemptive transplantation, children on dialysis for more than one year had a 52% higher risk of graft failure and those on dialysis for more than 18 months had an 89% higher risk of graft failure, regardless of donor source (living or deceased) [40]. In a further study of 1,113 pediatric kidney recipients, the relative risk of graft failure increased by 67% in children with more than two years of dialysis compared to children who underwent a preemptive transplantation [41]. These findings should be thoroughly explained to caregivers deciding to enlist their child on a deceased donor waiting list, and the advantage of an earlier transplantation should be considered together with the quality of the donor kidney.

The modality of dialysis, namely peritoneal dialysis or hemodialysis, prior to renal transplantation has also been reported to play an important role in posttransplant outcomes (in our study, only significant in the univariate analysis). In a recent systematic review and meta-analysis of 269,715 adult and pediatric patients, Ngamvichchukorn et al. demonstrated that peritoneal dialysis prior to renal transplantation was significantly associated with a lower risk of overall graft failure and a lower rate of delayed graft function [14]. It is believed that peritoneal dialysis provides a better fluid balance than hemodialysis and that hemodialysis may be associated with chronic volume deficiency or a higher proinflammatory state prior to transplantation, and studies in the adult population have significantly associated hemodialysis with adverse cardiovascular events following kidney transplantation [15,42,43]. The advantages of peritoneal dialysis were also confirmed in our study and hemodialysis in the univariate analysis was significantly associated with poorer graft survival outcomes compared to children having undergone peritoneal dialysis prior to kidney transplantation (*p* < 0.01 in the univariate analysis, not significant in the multivariate analysis) (Figure 3).

The main limitations of our study are its small size and the lack of consideration of further recipient- and donor-related baseline data, such as the cause for ESRD, the infection status of the recipient and the donor at transplantation, as well as the BSA of the donor. The small number in some subgroups, such as living donor recipients or transplantations involving a cold-ischemia time of more than 24 h, prohibited a comprehensive investigation of these subgroups. Furthermore, our study does not take into consideration events following transplantation that could very well influence long-term graft survival, such as the immunotherapy schemes used and posttransplant adherence to immunosuppressive medication, delayed graft function, or future infections, as well as the ultimate reason for graft failure. In consideration of the relatively limited number of pediatric kidney transplantations performed in a single center each year, we suggest that longitudinal multicenter studies be conducted to further evaluate other putative risk factors that could influence long-term kidney graft survival.

## 5. Conclusions

Despite its small size, our study confirms the excellent long-term graft survival rates of small patients with a BSA of <1 m^2^ (significant only in the univariate analysis), it shows an advantage of peritoneal dialysis prior to kidney transplantation compared to hemodialysis (significant only in univariate analysis) and it highlights the importance of donor/recipient age differences, as well as the importance of pretransplantation duration on dialysis in long-term kidney graft survival following pediatric kidney transplantation. Our study demonstrates that a donor/recipient age difference of ≥25 years and a pretransplantation duration on dialysis of ≥18 months are significantly associated with poorer long-term kidney graft survival, and both factors should be taken into consideration in the decision-making process during organ allocation.

## Figures and Tables

**Table 1:** Risk factor analysis of pediatric kidney transplantations over the last 20 years in our center (significant values are in bold face).

Parameter		Loss of Graft	Mean Graft Survival (Months)	Log-Rank *p* Value
Organ				*p* = 0.2
	Living donor (LD)	1/5	216	
	Postmortem (DD)	29/80	159	
Transplantation				*p* = 0.8
	Primary transplant	27/73	162	
	Retransplant	3/12	171	
Recipient sex				*p* = 0.9
	female	14/38	165	
	male	16/47	163	
Recipient age				***p* = 0.01**
	<12 years	11/43	185	
	≥12 years	19/42	139	
Donor age				***p* = 0.03**
	<35 years	8/40	194	
	≥35 years	21/42	145	
Donor sex				*p* = 0.5
	female	12/30	154	
	male	18/55	165	
Age difference				***p* = 0.04**
	<25 years	8/37	196	
	≥25 years	21/45	149	
Sex mismatch (donor/recipient)				*p* = 0.3
	Sex match	16/47	171	
	Sex mismatch	14/38	159	
Recipient BSA				***p* < 0.01**
	<1 m^2^	9/36	193	
	≥1 m^2^	21/49	139	
Recipient time on dialysis				***p* = 0.02**
	<18 months	4/22	206	
	≥18 months	26/63	147	
Modality of dialysis				***p* < 0.01 **
	peritoneal dialysis	11/43	180	
	hemodialysis	18/36	131	
HLA mismatches				*p* = 0.1
	<3	17/34	144	
	≥3	11/42	181	
Cold ischemia time				*p* = 0.6
	<12 hours	13/39	171	
	≥12 hours	17/46	157	

**Table 2:** Multivariate Cox regression survival analysis of risk factors for long-term kidney graft survival following pediatric kidney transplantation (significant values are in bold face).

Parameter		HR (95% CI)	*p* Value
Age difference			
	<25 years	reference	
	≥25 years	**3.039** (1.253–7.369)	**0.02**
Recipient BSA			
	<1 m^2^	reference	
	≥1 m^2^	1.753 (0.729–4.219)	0.21
Recipient time on dialysis			
	<18 months	reference	
	≥18 months	**3.869** (1.114–13.433)	**0.03**
Modality of dialysis			
	Peritoneal dialysis	reference	0.15
	Hemodialysis	1.864 (0.795–4.373)	

## Data Availability

All data are available in the manuscript. Detailed datasets used and analyzed during the present study are available from the corresponding author upon reasonable request.
